# Bioinspired Synthesis of ZnO@polydopamine/Au for Label-Free Photoelectrochemical Immunoassay of Amyloid-β Protein

**DOI:** 10.3389/fbioe.2021.777344

**Published:** 2021-11-16

**Authors:** Guangli He, Yue Zhou, Mifang Li, Yanzhen Guo, Hang Yin, Baocheng Yang, Shouren Zhang, Yibiao Liu

**Affiliations:** ^1^ Henan Key Laboratory of Nanocomposites and Applications, Institute of Nanostructured Functional Materials, Huanghe Science and Technology College, Zhengzhou, China; ^2^ Shenzhen Longgang Central Hospital (The Second Affiliated Hospital of the Chinese University of Hong Kong, Shenzhen, China

**Keywords:** photoelectrochemical sensor, ZnO, polydopamine, gold nanoparticles, Alzheimer’s disease

## Abstract

Amyloid-β protein (Aβ) is an important biomarker and plays a key role in the early stage of Alzheimer’s disease (AD). Here, an ultrasensitive photoelectrochemical (PEC) sensor based on ZnO@polydopamine/Au nanocomposites was constructed for quantitative detection of Aβ. In this sensing system, the ZnO nanorod array decorated with PDA films and gold nanoparticles (Au NPs) have excellent visible-light activity. The PDA film was used as a sensitizer for charge separation, and it also was used for antibody binding. Moreover, Au NPs were loaded on the surface of PDA film by *in situ* deposition, which further improved the charge transfer efficiency and the PEC activity in visible light due to the localized surface plasmon resonance effect of Au NPs. Therefore, in ZnO@polydopamine/Au nanocomposites, a significantly enhanced photocurrent response was obtained on this photoelectrode, which provides a good and reliable signal for early detection of AD. Under the optimized conditions, the PEC immunosensor displayed a wide linear range from 1 pg/mL to 100 ng/mL and a low detection limit of 0.26 pg/mL. In addition, this PEC immunosensor also presented good selectivity, stability, and reproducibility. This work may provide a promising point-of-care testing method toward advanced PEC immunoassays for AD biomarkers.

## Introduction

As a most prevalent type of dementia, Alzheimer’s disease (AD) is a fatal and irreversible neurodegenerative disorder, occurring mainly in aged people.(Evans, Funkenstein et al., 1989; Matthews, Arthur et al., 2013) Although massive effort has been made to cure this disease, there is still no efficient treatment for it. AD is marked by a slow degeneration progression and the neurodegeneration process starts several decades before the first clinical symptoms appear.(Jack et al., 2010; Selkoe 2012; Yang et al., 2021) Therefore, early diagnosis of AD allows timely treatment to ameliorate the deterioration symptoms of the patient, which has important directive significance to clinical works.(Butterfield, Drake et al., 2002; Hardy 2002; Jack et al., 2010; Murphy and Rd 2010) One of the important traits of AD is cerebral extracellular amyloid plaques, which are formed through aggregation of amyloid-β (Aβ) protein. Aβ protein is a polypeptide consisting of 39–42 amino acids. There are two primary variants: Aβ40 and Aβ42. PET and the level of Aβ (Aβ40, Aβ42) in cerebrospinal fluid (CSF) are the gold standard of AD clinical diagnosis.(Grimmer, Riemenschneider et al., 2009; Marcus, Mena et al., 2014; Guilherme, Marina et al., 2020) However, this diagnosis method is unlikely to become popularized in the public because of high-cost PET and unavailable CSF. Therefore, the development of low-cost, noninvasive, and accessible tools to accurately quantify Aβ protein in blood for the early diagnosis of AD is required.(Nakamura, Kaneko et al., 2018; Laís Canniatti, Isabella et al., 2019) In blood, the physiological concentrations of Aβ is several picograms per milliliter. To date, a great deal of sensing techniques has been performed to detect Aβ protein, including ELISA(Linan, Son et al., 2016), ion mobility-mass spectrometry (IM-MS)(Obata, Murakami et al., 2020), colorimetric biosensor(Xi, Shuangling et al., 2021), electrochemical(Carneiro, Loureiro et al., 2017), fluorescence(Lee, Park et al., 2019; Wang, Du et al., 2020; Wen-Kai, Liu et al., 2021), surface-enhanced Raman spectroscopy(Yang, Hwang et al., 2019), and photoelectrochemical (PEC) immunosensors(Wang, Fan et al., 2018). Among them, the PEC biosensor is an innovative and attractive analytical technique for quantitative study in the biological analysis due to its intrinsic merits such as label-free, high signal-to-noise ratio, rapid response, and is more readily miniaturized.(Shu and Tang 2019)

For the construction of the PEC biosensor, various kinds of semiconductor materials have been developed to construct the photoelectrode for PEC sensing. Zinc oxide (ZnO) is one of the most extensively used n-type semiconductor photoactive substrates with the advantages of environmental friendliness, abundant natural resources, low cost, and high stability.(Wei, Ke et al., 2012; Zhang, Wang et al., 2016) However, ZnO has the inherent limitations of inefficient utilization of sunlight and low separation efficiency of electron hole, which cannot meet the demands for higher sensitivity of PEC detection. To improve the light-harvesting efficiency and prolong the life of photo-generated carriers, combining with plasmon metals including Au, Ag, and Cu exhibits great superiority in improving the light absorption range and separation efficiency of charge carriers of ZnO-based systems over the other strategies due to the surface plasmon resonance (SPR) effect.(Zheng, Zheng et al., 2007; Xiao, Liu et al., 2015; Kang, Yan et al., 2016; Yang, Li et al., 2017; Zhang, He et al., 2018) Based on the localized surface plasmon resonance (LSPR) of Au nanoparticles (Au NPs), the strong visible light absorption and the effective photo-generated carrier separation at the metal–semiconductor interface can successfully generate remarkable readout photocurrent signals.(Zhu, Zhang et al., 2016; Zhang; Wang et al., 2018; Dong, Xu et al., 2019) For PEC biosensor, the efficient immobilization of biomolecules on photoactive materials of the photoelectrode is also a crucial factor for achieving excellent performance.(Shu and Tang 2019) For ZnO/Au substrates, the antibody immobilization is mainly through Au-NH_2_ bonding(Parviz Norouzi and Ganjali 2011; Gasparotto, Costa et al., 2017), but the amount of the immobilized antibody is limited by the low Au content. Recently, inspired by the mussel-adhesion phenomenon, polydopamine (PDA) has attracted intensive attention as a multifunctional biocompatible material with unique properties.(Lee, Dellatore et al., 2007; Lynge, van der Westen et al., 2011; Liu, Ai et al., 2014) Through self-polymerization of dopamine in alkaline aqueous solutions at room temperature, PDA can coat on the surface of various kinds of substrates with strong affinity. The as-formed adherent PDA thin film has abundant active functional groups (catechol, amine, indole, and quinone), capable to serve as universal platforms including cross-linker reagents for biomolecule immobilization and reductants for *in situ* metal deposition. Besides, PDA possesses broadband light absorption and can work as an electron donor.(Hu, He et al., 2014; Wang, Ma et al., 2017; Yu, Huang et al., 2018; He, Gao et al., 2019) Thus, PDA that is used to build up PEC immunosensor exhibits great advantages as a multifunctional platform.

**FIGURE 1:**
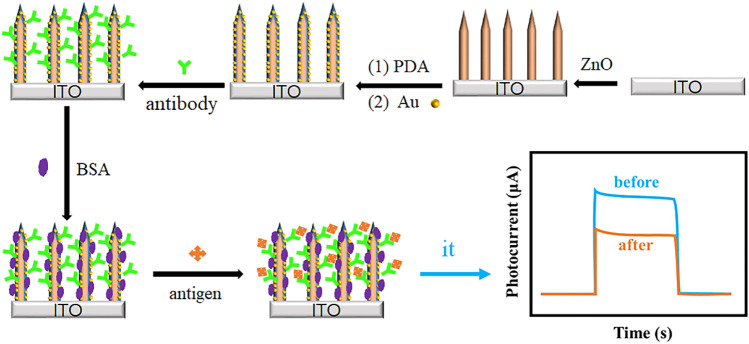
The stepwise fabrication process of the ZnO@PDA/Au PEC immunosensor.

In this work, a novel plasmonic PEC immunosensor was successfully constructed based on ZnO nanorod array@polydopamine heterointerface modified with Au NPs (ZnO@PDA/Au) for sensitive detection of Aβ. The stepwise process for the PEC immunosensor fabrication and the charge transfer mechanism are displayed in Figure 1. The PDA coating involving in the system is used as a multifunctional platform such as a sensitizer for enhancing the photo-to-electron conversion efficiency, a binder for antibody bonding, and a reductant for *in situ* Au deposition. Both PDA film and Au NPs can enhance the light absorption and loading content of antibody. The as-prepared PEC immunosensor realized label-free detection of Aβ, which exhibits a wide linear range from 1 pg/mL to 100 ng/mL, low detection limit of 0.26 pg/mL, high sensitivity, and good stability. This work provides a promising future for the early diagnosis of AD.

## Experimental Section

### Chemicals and Materials

Aβ40, Aβ42, and Aβ42 antibodies were purchased from Bio-Techne China Co., Ltd (Shanghai, China). Bovine serum albumin (BSA) was obtained from Kuer Chemical Technology (Beijing) Co., Ltd (Beijing, China). Zinc nitrate hexahydrate (Zn(NO_3_)_2_·6H_2_O), ammonia hydroxide (NH_3_·H_2_O), ethylenediamine, and potassium permanganate (KMnO_4_) were acquired from Sinopharm Chemical Reagent Co., Ltd (Shanghai, China). Dopamine, sodium chloride (NaCl), potassium chloride (KCl), Na_2_HPO_4_·12H_2_O, KH_2_PO_4_, and ascorbic acid (AA) were purchased from Aladdin Biochemical Technology Co., Ltd., (Shanghai, China). Indium-doped tin oxide (ITO) conducting glass (sheet resistance 8 Ω) was acquired from Wuhan Jinge Solar Technology Co., Ltd., (China). All the chemical reagents were of analytical grade and used directly as received. All the solutions used in all experiments were prepared with ultrapure water (Milli-Q, 18.2 MΩ).

### Characterization and Measurement

The morphology was characterized using field emission scanning electron microscopy (FESEM; Quanta 250, FEI, OR, United States). The energy-dispersive X-ray spectroscopy was used to reveal the elemental composition of all composites equipped with FESEM. The X-ray diffraction analyzer (XRD; Bruker D8 diffractometer, Germany) using Cu Kα radiation (40 kV, 40 mA) was employed to determine the crystal phase of the obtained samples. The UV–vis diffuse reflectance spectra (DRS) of the samples were collected using a Thermo U-4100 UV–vis spectrophotometer. The X-ray photoelectron spectroscopy (XPS) data of the composite was obtained on a Thermo Scientific K-Alpha X-ray photoelectron spectrometer with a monochromatized Mg Kα X ray source. All the electrochemical measurements were carried out on a CHI760E Electrochemical Analyzer (Chenhua Instruments, Shanghai, China).

### Preparation of ZnO@PDA/Au Modified ITO Electrode

The ZnO nanorod array was grown onto ITO substrate based on the previous literature.(Yang and Hu 2017; He, Gao et al., 2019) In brief, a piece of clean ITO (tailored as size of 2.5 × 1 cm) was first immersed into freshly prepared KMnO_4_ (5 mM) solution for 30 min at room temperature. After thoroughly rinsing with deionized water, the ITO was then placed with the conductive side facing down in a glass bottle with 10 mL precursor solution containing Zn(NO_3_)_2_ (0.1 M), ammonium hydroxide (3% v/v), and ethylenediamine (4% v/v). The reaction was carried out at 75°C in a water bath for 3 h. The obtained ZnO-ITO was rinsed with water and dried under gentle N_2_ flow for later use. Then, the ZnO-ITO electrodes were further coated with PDA *via* a simple dip-coating method. Typically, the as-synthesized ZnO-ITO were dipped into 20 mL of Tris–HCl solution (10 mM, pH 8.5) containing 25 mg dopamine and kept for 4 h at room temperature, followed by gently rinsing with DI water several times. The as-obtained ZnO@PDA electrodes were stored at 4°C for the following use. Au NPs decorated ZnO@PDA-ITO was obtained based on the following *in situ* growth method. Then 0.2 mL of HAuCl_4_ (1%) aqueous solution was added to 10 mL H_2_O solvent to obtain a homogeneous solution. Then the prepared ZnO@PDA-ITO were immersed into the solution and incubated for 60 min. These were then taken out and rinsed gently with DI water and stored at 4°C for next use.

### Construction of the Label-free PEC Immunosensors

First, the ZnO@PDA/Au electrode was incubated with 0.02 mL anti-Aβ (10 μg mL^−1^) PBS solution (pH = 7.4) on the electrode surface for 1 h at room temperature. Second, the electrode was rinsed with PBS solution to remove the physical adsorption of antibodies. Third, the electrode was dipped into bovine serum albumin (BSA, 1 mg/mL) PBS solution for 1 h to block the non-specific binding sites. Finally, 10 μL of Aβ with different concentrations was dropped onto the electrode surface and incubated 1 h at room temperature. After thoroughly washing with PBS buffer, the sensors used for the following PEC detection were obtained.

### Photoelectrochemical Detection

All the PEC tests were performed on a CHI 760E electrochemical workstation adopting a standard three-electrode system, in which the as-prepared photo-electrode was used as the working electrode, platinum (Pt) sheet as the counter electrode, and saturated Ag/AgCl electrode as the reference electrode. A 500-W Xe lamp equipped with an AM 1.5 G filter was employed as the irradiation source for the photo-response of the as-obtained photoelectrodes. The power density is 100 mW cm^−2^. 25 mL of PBS (0.1 M, pH 7.4) solution containing 1 mM AA served as the supporting electrolyte. Linear sweep voltammetry (LSV) curves were recorded from −0.05 to 0.3 V with a scan rate of 50 mV s^−1^. Electrochemical impedance spectroscopy (EIS) experiment was carried out at open circuit potential in the frequency range of 100 kHz to 0.1 Hz with an amplitude of 5 mV. The photocurrent response measurements of the working electrode were collected under light irradiation switching on and off every 10 s at the applied potential of 0.05 V.

## Results and Discussion

### Characterization of the as-prepared Electrodes

**FIGURE 2:**
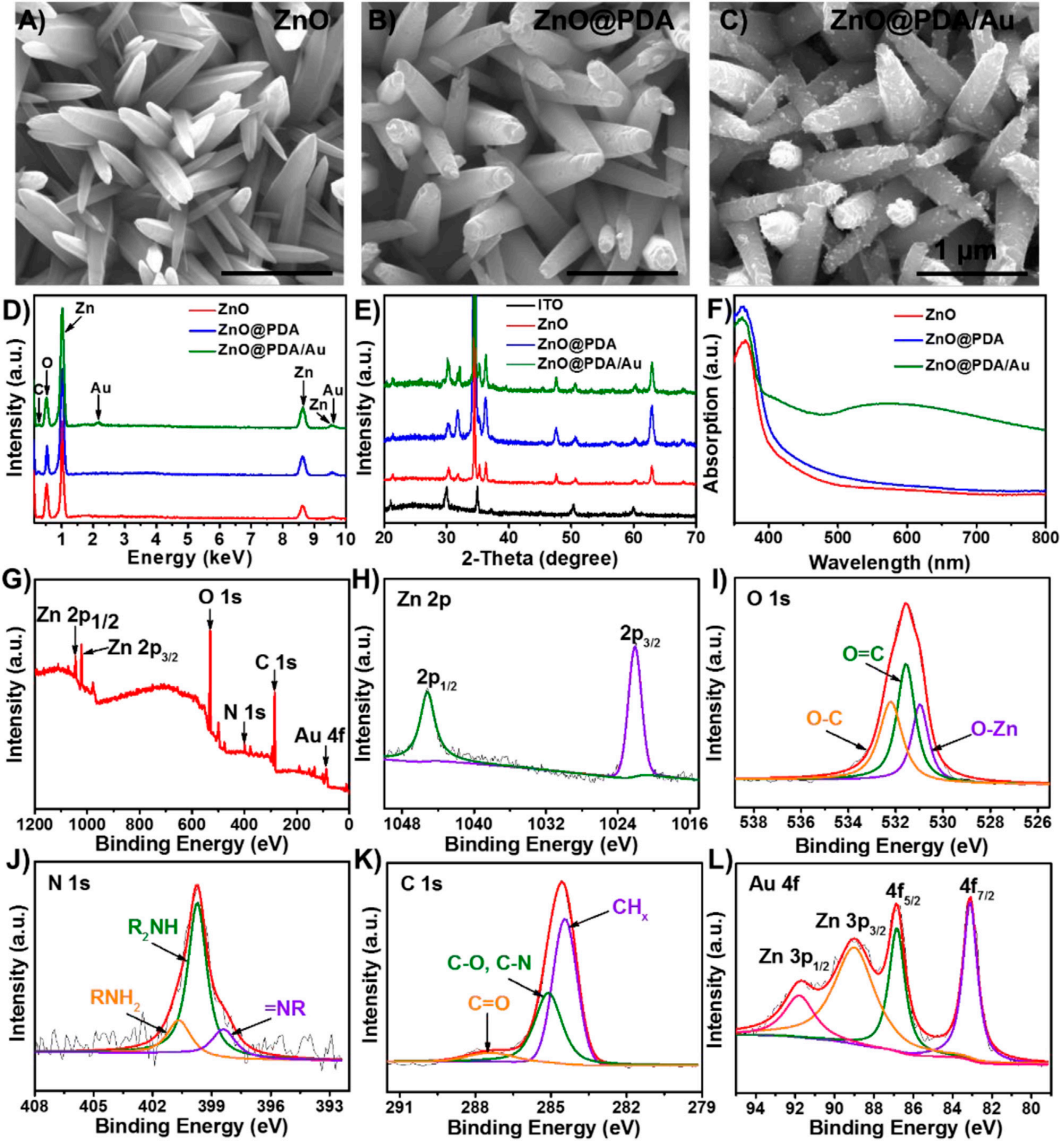
SEM images of **(A)** ZnO NRs, **(B)** ZnO@PDA, **(C)** ZnO@PDA/Au, and **(D)** EDS spectrum of ZnO NRs, ZnO@PDA and ZnO@PDA/Au. **(E)** XRD pattern of ITO, ZnO NRs, ZnO@PDA, and ZnO@PDA/Au. **(F)** UV–vis absorption spectra for ZnO NRs, ZnO@PDA, and ZnO@PDA/Au. XPS spectra of ZnO@PDA/Au. **(G)** Survey; (**H**) Zn 2p; **(I)** O 1s; **(J)** N 1s; **(K)** C 1s; **(L)** Au 4f and Zn 3p.

The morphology, structure, and composition of ZnO, ZnO@PDA, and ZnO@PDA/Au modified electrodes were recorded. The top and cross-sectional view SEM images in Figure 2A; Supplementary Figure S1 display that a large scale of ZnO nanorods with lengths of around 1.2 μm and diameters of less than 200 nm are grown on the ITO glass. The one-dimensional structure and large gap between the ZnO nanorods can provide a high surface area to facilitate light harvest and further biomaterial assembly. As shown in Figure 2B; Supplementary Figure S2A, after coating with PDA, the surface of modified nanorods become rougher compared with that of bare ZnO nanorods indicating that the ZnO nanorods have been successfully enwrapped by PDA to form a core/shell. The original structure of the ZnO nanorods still remained. The color of the substrate turns obviously from white to dark yellow during this process, as shown in Supplementary Figure S3. The ZnO@PDA were further used for *in situ* deposition of Au NPs basing on the reducing action of PDA coating. In Figure 2C; Supplementary Figure S2B, various Au NPs are observed homogeneously adhering on the surfaces of ZnO@PDA. At this time, the color of the electrode turned dark red (Supplementary Figure S3). EDS spectrum was conducted to further analyze the elemental composition of the electrode with different modifications. The results in Figure 1D exhibit that the ZnO nanorods mainly contain Zn and O elements. The content of the C element increases after PDA coating providing another evidence for the existence of PDA. The distinctive Au signal of the ZnO@PDA/Au further confirms that Au has been introduced to the coating substrate successfully. Figure 2E shows the XRD patterns for the structural analysis of all the samples. The ZnO exhibits sharp diffraction peaks at 31.7°, 34.5°, 36.3°, 47.6°, and 62.9°, which could be assigned to the hexagonal ZnO phase (JCPDS No. 70–2551). Compared with the original ZnO, PDA-coated ZnO show no peak positions altering and additional peaks, indicating that wrapping PDA does not affect its crystal phase, while a minor increase of the peak intensity can be seen, which might be owing to the low content of PDA in the complex and the positive effect of PDA on the crystallization of ZnO. After loading Au NPs, the emerging characteristic peak of Au at 38.4° proves that Au NPs exist in the ZnO@PDA/Au composite.

**FIGURE 3:**
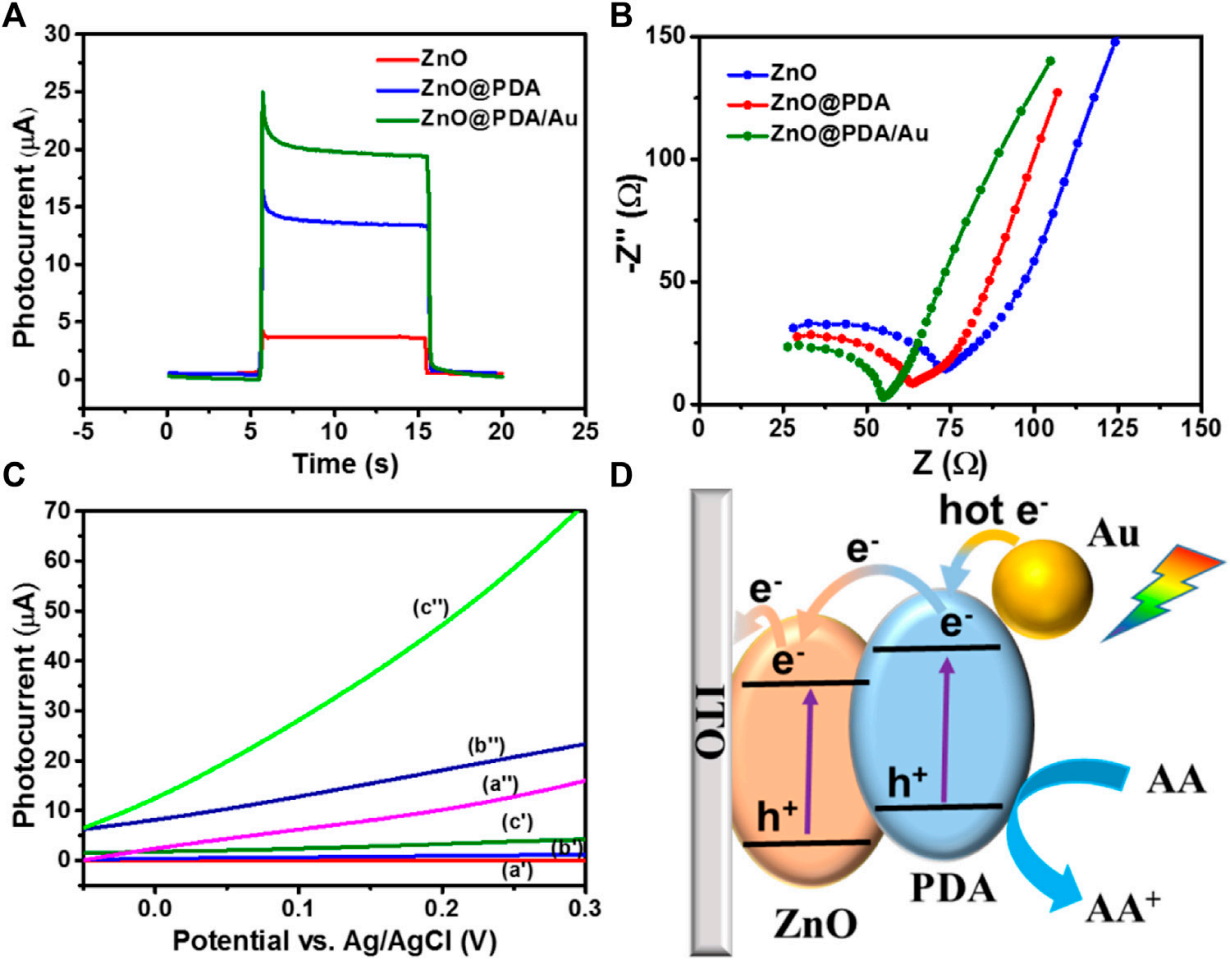
**(A)** Photocurrent response of ZnO NRs/ITO, ZnO@PDA, and ZnO@PDA/Au toward on/off cycles of simulated sunlight illumination at 0.1 V. Electrolyte solution was 0.1 M PBS containing 1 mM ascorbic acid, potential sweep rate at 50 mV s^−1^. **(B)** EIS Nyquist plots spectra of different modified electrodes ZnO NRs/ITO, ZnO@PDA, and ZnO@PDA/Au. **(C)** LSV curves of ZnO NRs/ITO (a′, a″), ZnO@PDA/ITO (b′, b″), and ZnO@PDA/Au/ITO (c′, c″) without/with simulated sunlight illumination, respectively. **(D)** The charge transfer mechanism of this PEC immunosensor.

Also, the optical properties of these samples were determined by UV–vis diffuse reflectance spectra (DRS). As presented in Figure 2F, the light absorbance of bare ZnO is weak in the visible region, which is ascribed to its intrinsic wide bandgap. However, owing to the homogeneous coating of the PDA shell, ZnO@PDA shows an extended absorption range to the visible light region and increased absorption intensity, indicating that PDA can efficiently absorb visible light and works as a photosensitizer to improve the charge separation. The ZnO@PDA/Au exhibits an obvious absorption peak at about 560 nm, which is assigned to the SPR peak of Au NPs. The aforementioned results prove that by combining the PDA core–shell heterostructure and the LSPR effect of Au NPs, more photoelectric carriers can be generated and separated, which plays a crucial role in enhancing the utilization of sunlight for PEC sensing. In addition, the XPS spectrum is used to analyze the chemical composition and electronic structures of ZnO@PDA/Au. The XPS survey spectrum (Figure 2G) shows the existence of Zn, O, N, C, and Au elements in the hybrid composite. As displayed in Figure 2H, two peaks of Zn 2p at about 1,022.1 and 1,045.2 eV can be indexed to the binding energy of Zn 2p_3/2_ and Zn 2p_1/2_, respectively. In Figure 2I, the O 1s peak is split into three peaks at 530.8, 531.5, and 532.3 eV, assigning to O–Zn bond binding energy in the structure of ZnO, O=C, and O–C in the PDA film, respectively (*Applied Surface Science* 457 (2018) 1096). As for the N 1s spectrum (Figure 3J), the three deconvoluted peaks centered at 398.4, 399.7, and 400.8 eV can be ascribed to primary (R = NH_2_), secondary (R−NH−R), and tertiary/aromatic (=N−R) amine functional groups in PDA, respectively. The C 1s spectrum (Figure 3K) exhibits three characteristic peaks at 284.4 eV for CH_x_ species, 285.1 eV for C–O/C–N, and 287.5 eV for C=O in PDA. In Figure 3L, the XPS spectrum of Au 4f strongly overlaps with the peaks of Zn 3p; thus, it was fitted into four peaks. The two peaks with the lower binding energy located in 83.1 and 86.8 eV have corresponded to the Au 4f_7/2_ and Au 4f_5/2_, respectively. Meanwhile, another two peaks can be indexed to Zn 3p_3/2_ (89.1 eV) and Zn 3p_1/2_ (91.9 eV), respectively. Therefore, the aforementioned findings confirm the successful synthesis of ZnO@PDA/Au.

### Photoelectrochemical behaviors and charge transfer mechanism of the PEC immunosensor

Transient photocurrent, electrochemical impedance analysis (EIS), and linear sweep voltammograms (LSVs) were applied to estimate intrinsic electrochemical behavior and photocurrent response in the ZnO@PDA/Au system. The transient photocurrent responses of the as-fabricated electrodes have been measured at 0.05 V (Figure 3A). The ZnO@PDA/Au electrode owns the highest photocurrent, which is about fivefold of the pristine ZnO. The excellent PEC performance of ZnO@PDA/Au benefits from the synergistic action of PDA and Au NPs, which broaden the light absorption range and enhance the separation efficiency of photo-generated electron–hole pairs. Moreover, EIS was used to test the electron transfer resistance change of different modification processes. As shown in Figure 3B, compared with ZnO and ZnO@PDA, the ZnO@PDA/Au shows the smallest interfacial electron transfer resistance.

The LSV curves of the ZnO, ZnO@PDA, and ZnO@PDA/Au electrodes were recorded in dark and under illumination with the applied potential ranging between −0.05 and 0.3 V. As shown in Figure 3C, all the as-prepared photoelectrodes display negligible current in the dark condition although the current density of ZnO@PDA/Au is slightly higher than the other two electrodes. Under irradiation, the PDA-coated ZnO displays a higher photocurrent than that of the original ZnO. This increase of photocurrent is attributed to the enhanced light harvest capability by using PDA as the photosensitizer. Obviously, after the subsequent decoration of Au NPs on the ZnO@PDA, the photocurrent significantly increases under the same potential because of higher light absorption and the photocarriers’ generation ability of Au nanoparticles. As a control, LSV curves of ITO are presented without/with simulated sunlight illumination, respectively, and the result is shown in Supplementary Figure S4A. Moreover, photocurrent responses of ITO, ITO/anti-Aβ/BSA, and ITO/anti-Aβ/BSA/Aβ toward on/off cycles of simulated sunlight illumination at 0.1 V were also performed as shown in Supplementary Figure S4B. These results demonstrated that ITO has no PEC performance. According to the aforementioned analysis, the decoration of PDA and Au NPs on ZnO can bring excellent PEC performance.

According to the aforementioned results, the possible photo-generated charge transfer mechanism of the ZnO@PDA/Au is illustrated in Figure 3D. Under the solar light illumination, each component of the as-prepared photoanode could be excited to generate carriers simultaneously. Light irradiation of the ZnO induces the excited electrons from the valence band (VB) to the conduction band (CB) and further transfer to the surface of ITO. At the interface of PDA and ZnO, the generated electrons on the lowest unoccupied molecular orbital (LUMO) of PDA could transfer to the CB of ZnO to generate a built-in electric field due to the matching energy level, which effectively blocks the photo-generated charge recombination and enhance the charge separation and transfer efficiency. In addition, the plasmonic Au NPs could generate hot electrons with sufficient energy and inject into the LUMO of PDA under visible light excitation. The enriched electrons on the ZnO would transfer to the counter Pt photocathode through an external circuit to obtain the photocurrent signal for the PEC detection. Meanwhile, the remaining holes on the VB of ZnO transfer to the highest occupied molecular orbital of PDA. Both the accumulated holes on PDA and hot holes left on the Au NPs can be rapidly consumed by AA (electron donor) in the electrolyte against the photoinduced carrier recombination. Accordingly, by integrating the photosensitization of PDA and LSPR effect of Au NPs, the ZnO@PDA/Au is considered to be an excellent substrate material for photocurrent response.

### Optimization of Detection Conditions

To obtain the best detection performance of photoelectrode, the AA concentration and the pH values, which have a direct effect on PEC response signal through electron transfer, were optimized in detail. AA can be used as the scavenger to consume the photoinduced hole and an efficient antioxidant to reduce the photo-oxidized PDA film. The change of photocurrent with the AA concentration range from 0.01 to 100 mM is shown in Supplementary Figure S5A. The photocurrent signal of the ZnO@PDA/Au electrode displays a significant increment along with the increasing AA concentration at first and then reaches a peak at 1 mM. A decrease in photocurrent density can be seen, when the AA concentration in the solution continues to increase. When the photo-generated electrons are injected into ZnO, the PDA and Au NPs could be regenerated through gaining electrons from the electron donor to achieve effective separation of photo-generated charge. At the same time, the excess AA in the electrolyte solution leads to the quenching absorption of the solution, which impacts the irradiation reaching the photoanode and reduces the intensity and efficiency of the excitation electron-hole center. Thus, 1 mM was selected as the optimal concentration of AA in this work.

Furthermore, the effect of pH value on the detection photocurrent was recorded in Supplementary Figure S5B. The photocurrent gradually increases with the pH of the buffer solution rising from 6.5 to 7.4. When the pH value further enhances to 8.5, the photocurrent shows an obvious decrement. This result may be ascribed to the bioactivity of the immobilized antigen that could be disrupted in the acidic or alkaline environment. The PBS solution with pH 7.4 is similar to the physiological environment to maintain the good activity of the biomolecules. Thus, to obtain an excellent photocurrent response and immunocomplex layer on the photoanode, pH 7.4 was selected in the following detection. In addition, the concentration of antibody was optimized, and the result shows the optimal concentration is 10 μg/mL (Supplementary Figure S6).

### Analytical Performance of the Photoelectrochemical Immunosensor

Based on the aforementioned optimal conditions, the photocurrent density was collected to track the succession process for the fabrication of the PEC immunosensor. In Supplementary Figure S7, after the immobilization of anti-Aβ (curve b) and subsequent BSA blocking (curve c), the photocurrent intensity reduces significantly. This is ascribed to the binding molecules on the surface of the photoanode that partly hinder the access and reaction of AA with the photoinduced holes. The further decrease of photocurrent can be seen after continued incubation with Aβ, owing to the formation of the antigen–antibody immunocomplexes on the photoelectrode surface. These results confirm the successful construction of the label-free PEC immunosensor.

**FIGURE 4:**
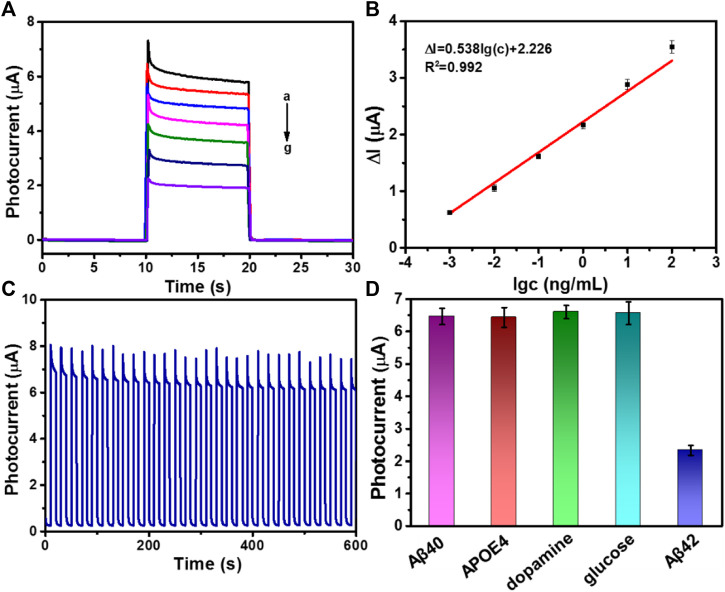
**(A)** Photocurrent responses of Aβ with different concentration, from (**a**) to (**g**): 0, 0.001, 0.01, 0.1, 1, 10, and 100 ng/mL. **(B)** The linear calibration curve for photocurrent densities versus various concentrations of Aβ (Δ*I* = *I*_0_ − *I*, *I*_0_ is the photocurrent response of the PEC sensor without Aβ and *I* is the photocurrent response containing antigen). Error bars = RSD (*n* = 3). **(C)** Stable photocurrent response curve of ZnO@PDA/Au electrode incubated with 1 pg/mL Aβ. **(D)** Selectivity of the PEC immunosensor. Photocurrent responses of the PEC immunosensor for different proteins including 1000 pg/mL Aβ40, 1,000 pg/mL APOE4, 1,000 pg/mL dopamine, 1,000 pg/mL glucose, or 10 pg/mL Aβ42.

The photocurrent intensity has a direct relationship with the concentration of Aβ. Thus, the as-fabricated immunosensor based on ZnO@PDA/Au was applied to detect Aβ with different concentrations. Figure 4A displays the photocurrent change of the as-fabricated PEC biosensor with different concentrations of Aβ at the optimal condition. It can be seen that along with the increase of the Aβ concentration, the photocurrent reduces gradually. The linear relationship related to the change of the photocurrent intensity (Δ*I*) and the logarithm of Aβ concentration in the range from 1 pg mL^−1^ to 100 ng mL^−1^ is presented in Figure 4B. The regression equation is displayed as Δ*I* = 0.538 log *c* + 2.226 (*R* ^2^ = 0.992). The limit of detection (LOD) is determined to be 0.26 pg mL^−1^ (S/N = 3). The LOD is calculated by three times the SD of the blank according to our previous report.(Yibiao, Qing et al., 2021) As a control, a typical ELISA has been used to validate our method. As shown in Supplementary Figure S8, the ELISA calibration curve was constructed by plotting the optical density of Aβ42 at 450 nm (TMB as substrate). And the range of Aβ42 concentration is 1–800 pg/mL. The relationship between the optical density at 450 nm and the Aβ42 concentration follows the regression equation *y* = 0.00099*x* + 0.072 (*R* ^2^ = 0.9991). The detection limit is 18.59 pg/mL, and the linear range is from 10 to 800 pg/mL. Compared with typical ELISA, our prepared PEC sensor based on ZnO@PDA/Au exhibited lower LOD and wider linear range. Compared with several previous reports for the detection of Aβ, the proposed PEC immunosensor in our work exhibits a much wider sensing range and competitive LOD. A comparison is shown in Supplementary Table S1. What is more, the as-fabricated material is environment friendly and possesses good biocompatibility. Therefore, the ZnO@PDA/Au is a very promising material used for the construction of sensitive PEC immunosensors.

### Stability, Reproducibility, and Specificity

The stability, reproducibility, and specificity are very crucial factors for the successful construction of immunosensor in biological application. The photocurrent response of as-prepared immunosensor incubated with 1 pg mL^−1^ Aβ was tested by 30 on/off cycles of illumination to access its stability. Observing from Figure 4C, the photocurrent response shows no distinct variation. Moreover, the as-fabricated immunosensor was stored at 4°C to investigate the storage stability. After 2 weeks of restoring, the photocurrent density could still retain 90.1% of its initial response (Supplementary Figure S9). In addition, the reproducibility of this ZnO@PDA/Au electrode was also measured, and the result is shown in Supplementary Figure S10, which demonstrated that the ZnO@PDA/Au electrode had excellent reproducibility. To evaluate the specificity of the fabricated PEC biosensor, several biological interfering species including Aβ40, human apolipoprotein E4 (APOE4), dopamine, and glucose with a 100-fold higher concentration were chosen for the test. As shown in Figure 4D, the photocurrent response to the interfering substances appears imperceptible, compared with that of the specific target Aβ42, which implied that the PEC immunosensor possesses excellent anti-interference ability and specificity. The aforementioned results prove that this proposed sensing platform owns superior stability, reproducibility, and specificity.

### Aβ Detection in Serum Sample

To further certify the practicability of the prepared PEC immunosensor, the tests were also carried out in real biological samples. The diluted serum samples were spiked with different concentrations of Aβ42(1, 10, 100, 1,000, 10,000 pg/mL), and then the photocurrent changes were recorded in Supplementary Table S2. The detection results are close to that of the added values with satisfactory recoveries ranging from 97.1 to 109.1%, revealing that the proposed PEC immunosensor based on ZnO@PDA/Au has excellent sensitivity and reliability for the detection of Aβ in real serum sample analysis and can be capable for the clinical diagnosis.

## Conclusion

In summary, a novel PEC immunosensor based on PDA and Au NP co-decorated ZnO was successfully designed for ultrasensitive and label-free detection of Aβ. The ZnO@PDA/Au substrate obtained by self-polymerization and *in situ* self-reduction exhibits superior optoelectronic property by taking the advantage of the photosensitization of PDA and SPR effect of Au NPs together. What is more, the PDA film and Au NPs can also be used as a biocompatible functional layer for the immobilization of biomolecules. The interfacial charge transfer mechanism of the proposed biosensing platform was investigated in detail. The as-fabricated PEC immunosensor displays apparent merits for Aβ detection including broad linear range, low detection limit, and good anti-interference performance and stability. In addition, it can also realize the acceptable quantitative analysis of Aβ in real serum sample. Due to the aforementioned advantages, the PEC electrode provides a versatile sensing platform and has good potential in other bioanalysis applications for early disease diagnosis.

## Data Availability

The original contributions presented in the study are included in the article/Supplementary Material, further inquiries can be directed to the corresponding authors.

## References

[B1] Brazaca L. C. Sampaio I. Zucolotto V. Janegitz B. C. (2020). Applications of Biosensors in Alzheimer's Disease Diagnosis. Talanta 210, 120644. 10.1016/j.talanta.2019.120644 31987214

[B2] Butterfield D. A. Drake J. Pocernich C. Castegna A. (2002). Evidence of Oxidative Damage in Alzheimer's Disease Brain: central Role for Amyloid Beta-Peptide. Trends Mol. Med. 7, 548–554. 10.1016/S1471-4914(01)02173-6 11733217

[B3] Carneiro P. Loureiro J. Delerue-Matos C. Morais S. do Carmo Pereira M. (2017). Alzheimer's Disease: Development of a Sensitive Label-free Electrochemical Immunosensor for Detection of Amyloid Beta Peptide. Sensors Actuators B: Chem. 239, 157–165. 10.1016/j.snb.2016.07.181

[B4] Dong X. Xu C. Yang C. Chen F. Manohari A. G. Zhu Z. (2019). Photoelectrochemical Response to Glutathione in Au-Decorated ZnO Nanorod Array. J. Mater. Chem. C 7, 5624–5629. 10.1039/c9tc00901a

[B5] Evans D. A. Funkenstein H. H. Albert M. S. Scherr P. A. Cook N. R. Chown M. J. (1989). Prevalence of Alzheimer's Disease in a Community Population of Older Persons. Jama 262, 2551–2556. 10.1001/jama.1989.03430180093036 2810583

[B6] Fang W.-K. Liu L. Zhang L.-l. Liu D. Liu Y. Tang H.-W. (2021). Detection of Amyloid β Oligomers by a Fluorescence Ratio Strategy Based on Optically Trapped Highly Doped Upconversion Nanoparticles-SiO2@Metal-Organic Framework Microspheres. Anal. Chem. 93, 12447–12455. 10.1021/acs.analchem.1c02679 34449219

[B7] Folego G. Weiler M. Casseb R. F. Pires R. Rocha A. (2020). Alzheimer's Disease Detection through Whole-Brain 3D-CNN MRI. Front. Bioeng. Biotechnol. 8. 10.3389/fbioe.2020.534592 PMC7661929 33195111

[B8] Gasparotto G. Costa J. P. C. Costa P. I. Zaghete M. A. Mazon T. (2017). Electrochemical Immunosensor Based on ZnO Nanorods-Au Nanoparticles Nanohybrids for Ovarian Cancer Antigen CA-125 Detection. Mater. Sci. Eng. C 76, 1240–1247. 10.1016/j.msec.2017.02.031 28482492

[B9] Grimmer T. Riemenschneider M. Förstl H. Henriksen G. Klunk W. E. Mathis C. A. (2009). Beta Amyloid in Alzheimer's Disease: Increased Deposition in Brain Is Reflected in Reduced Concentration in Cerebrospinal Fluid. Biol. Psychiatry 65, 927–934. 10.1016/j.biopsych.2009.01.027 19268916 PMC2700302

[B10] Hardy J. Selkoe D. J. (2002). The Amyloid Hypothesis of Alzheimer's Disease: Progress and Problems on the Road to Therapeutics. Science 297, 353–356. 10.1126/science.1072994 12130773

[B11] He G. Gao F. Li W. Li P. Zhang X. Yin H. (2019). Electrochemical Sensing of H2O2 Released from Living Cells Based on AuPd alloy-modified PDA Nanotubes. Anal. Methods 11, 1651–1656. 10.1039/C8AY02743A

[B12] Hu W. He G. Zhang H. Wu X. Li J. Zhao Z. (2014). Polydopamine-Functionalization of Graphene Oxide to Enable Dual Signal Amplification for Sensitive Surface Plasmon Resonance Imaging Detection of Biomarker. Anal. Chem. 86, 4488–4493. 10.1021/ac5003905 24712824

[B13] Jack C. R. Knopman D. S. Jagust W. J. Shaw L. M. Aisen P. S. Weiner M. W. (2010). Hypothetical Model of Dynamic Biomarkers of the Alzheimer's Pathological cascade. Lancet Neurol. 9, 4–5. 10.1016/S1474-4422(09)70299-6 20083042 PMC2819840

[B14] Kang Z. Yan X. Wang Y. Zhao Y. Bai Z. Liu Y. (2016). Self-powered Photoelectrochemical Biosensing Platform Based on Au NPs@ZnO Nanorods Array. Nano Res. 9, 344–352. 10.1007/s12274-015-0913-9

[B15] Lee H. Dellatore S. M. Miller W. M. Messersmith P. B. (2007). Mussel-Inspired Surface Chemistry for Multifunctional Coatings. Science 318, 426–430. 10.1126/science.1147241 17947576 PMC2601629

[B16] Lee S.-C. Park H.-H. Kim S.-H. Koh S.-H. Han S.-H. Yoon M.-Y. (2019). Ultrasensitive Fluorescence Detection of Alzheimer's Disease Based on Polyvalent Directed Peptide Polymer Coupled to a Nanoporous ZnO Nanoplatform. Anal. Chem. 91, 5573–5581. 10.1021/acs.analchem.8b03735 30938150

[B17] Liu Y. Ai K. Lu L. (2014). Polydopamine and its Derivative Materials: Synthesis and Promising Applications in Energy, Environmental, and Biomedical Fields. Chem. Rev. 114, 5057–5115. 10.1021/cr400407a 24517847

[B18] Liu Y. Xu Q. Zhang Y. Ren B. Huang L. Cai H. (2021). An Electrochemical Aptasensor Based on AuPt alloy Nanoparticles for Ultrasensitive Detection of Amyloid-β Oligomers. Talanta 231, 122360. 10.1016/j.talanta.2021.122360 33965026

[B19] Lynge M. E. van der Westen R. Postma A. Städler B. (2011). Polydopamine-a Nature-Inspired Polymer Coating for Biomedical Science. Nanoscale 3, 4916–4928. 10.1039/C1NR10969C 22024699

[B20] Marcus C. Mena E. Subramaniam R. M. (2014). Brain PET in the Diagnosis of Alzheimer's Disease. Clin. Nucl. Med. 39, e413–6. 10.1097/RLU.0000000000000547 25199063 PMC4332800

[B21] Matthews F. E. Arthur A. Barnes L. E. Bond J. Jagger C. Robinson L. (2013). A Two-Decade Comparison of Prevalence of Dementia in Individuals Aged 65 Years and Older from Three Geographical Areas of England: Results of the Cognitive Function and Ageing Study I and II. The Lancet 382, 1405–1412. 10.1016/S0140-6736(13)61570-6 PMC3906607 23871492

[B22] Murphy M. P. LeVine H. (2010). Alzheimer's Disease and the Amyloid-β Peptide. Jad 19, 311–323. 10.3233/jad-2010-1221 20061647 PMC2813509

[B23] Nakamura A. Kaneko N. Villemagne V. L. Kato T. Doecke J. Doré V. (2018). High Performance Plasma Amyloid-β Biomarkers for Alzheimer's Disease. Nature 554, 249–254. 10.1038/nature25456 29420472

[B24] Norouzi P. Gupta V. K. Faridbod F. Pirali-Hamedani M. Larijani B. Ganjali M. R. (2011). Carcinoembryonic Antigen Admittance Biosensor Based on Au and ZnO Nanoparticles Using FFT Admittance Voltammetry. Anal. Chem. 83, 1564–1570. 10.1021/ac102270w 21309514

[B25] Obata Y. Murakami K. Kawase T. Hirose K. Izuo N. Shimizu T. (2020). Detection of Amyloid β Oligomers with RNA Aptamers in AppNL-G-F/NL-G-F Mice: A Model of Arctic Alzheimer's Disease. ACS Omega 5, 21531–21537. 10.1021/acsomega.0c02134 32905362 PMC7469371

[B26] Selkoe D. J. (2012). Preventing Alzheimer's Disease. Science 337, 1488–1492. 10.1126/science.1228541 22997326

[B27] Shu J. Tang D. (2019). Recent Advances in Photoelectrochemical Sensing: From Engineered Photoactive Materials to Sensing Devices and Detection Modes. Anal. Chem. 92, 363–377. 10.1021/acs.analchem.9b04199 31738852

[B28] Song L. Lachno D. R. Hanlon D. Shepro A. Jeromin A. Gemani D. (2016). A Digital Enzyme-Linked Immunosorbent Assay for Ultrasensitive Measurement of Amyloid-β 1-42 Peptide in Human Plasma with Utility for Studies of Alzheimer's Disease Therapeutics. Alz Res. Ther. 8, 58–64. 10.1186/s13195-016-0225-7 PMC5160015 27978855

[B29] Wang R. Ma H. Zhang Y. Wang Q. Yang Z. Du B. (2017). Photoelectrochemical Sensitive Detection of Insulin Based on CdS/polydopamine Co-sensitized WO 3 Nanorod and Signal Amplification of Carbon Nanotubes@polydopamine. Biosens. Bioelectron. 96, 345–350. 10.1016/j.bios.2017.05.029 28525853

[B30] Wang X. Z. Du J. Xiao N. N. Zhang Y. Fei L. LaCoste J. D. (2020). Driving Force to Detect Alzheimer's Disease Biomarkers: Application of a Thioflavine T@Er-MOF Ratiometric Fluorescent Sensor for Smart Detection of Presenilin 1, Amyloid β-protein and Acetylcholine. Analyst 145, 4646–4663. 10.1039/D0AN00440E 32458857

[B31] Wang Y. Fan D. Zhao G. Feng J. Wei D. Zhang N. (2018). Ultrasensitive Photoelectrochemical Immunosensor for the Detection of Amyloid β-protein Based on SnO2/SnS2/Ag2S Nanocomposites. Biosens. Bioelectron. 120, 1–7. 10.1016/j.bios.2018.08.026 30142477

[B32] Wei Y. Ke L. Kong J. Liu H. Jiao Z. Lu X. (2012). Enhanced Photoelectrochemical Water-Splitting Effect with a Bent ZnO Nanorod Photoanode Decorated with Ag Nanoparticles. Nanotechnology 23, 235401. 10.1088/0957-4484/23/23/235401 22609803

[B33] Xiao S. Liu P. Zhu W. Li G. Zhang D. Li H. (2015). Copper Nanowires: A Substitute for Noble Metals to Enhance Photocatalytic H2 Generation. Nano Lett. 15, 4853–4858. 10.1021/acs.nanolett.5b00082 26189663

[B34] Yang J. K. Hwang I. J. Cha M. G. Kim H. I. Yim D. Jeong D. H. (2019). Reaction Kinetics‐Mediated Control over Silver Nanogap Shells as Surface‐Enhanced Raman Scattering Nanoprobes for Detection of Alzheimer's Disease Biomarkers. Small 15, 1900613. 10.1002/smll.201900613 30957959

[B35] Yang X. Li H. Zhang W. Sun M. Li L. Xu N. (2017). High Visible Photoelectrochemical Activity of Ag Nanoparticle-Sandwiched CdS/Ag/ZnO Nanorods. ACS Appl. Mater. Inter. 9, 658–667. 10.1021/acsami.6b12259 27982560

[B36] Yang Y. Hu W. (2017). Bifunctional Polydopamine Thin Film Coated Zinc Oxide Nanorods for Label-free Photoelectrochemical Immunoassay. Talanta 166, 141–147. 10.1016/j.talanta.2017.01.024 28213214

[B37] Yang Z. Wang Y. Zhang D. (2021). 2021 Alzheimer's Disease Facts and Figures. Alzheimer's Demen. 17, 327–406. 10.1002/alz.12328 33756057

[B38] Yu Y. Huang Z. Zhou Y. Zhang L. Liu A. Chen W. (2019). Facile and Highly Sensitive Photoelectrochemical Biosensing Platform Based on Hierarchical Architectured Polydopamine/Tungsten Oxide Nanocomposite Film. Biosens. Bioelectron. 126, 1–6. 10.1016/j.bios.2018.10.026 30388548

[B39] Zhang B. Wang F. Zhu C. Li Q. Song J. Zheng M. (2016). A Facile Self-Assembly Synthesis of Hexagonal ZnO Nanosheet Films and Their Photoelectrochemical Properties. Nano-micro Lett. 8, 137–142. 10.1007/s40820-015-0068-y PMC6223674 30460273

[B40] Zhang B. Wang H. Ye H. Xu B. Zhao F. Zeng B. (2018). Reversible Redox Mechanism Based Synthesis of Plasmonic WO3/Au Photocatalyst for Selective and Sensitive Detection of Ultra-micro Hg2+. Sensors Actuators B: Chem. 273, 1435–1441. 10.1016/j.snb.2018.07.070

[B41] Zhang Y. He S. Guo W. Hu Y. Huang J. Mulcahy J. R. (2018). Surface-Plasmon-Driven Hot Electron Photochemistry. Chem. Rev. 118, 2927–2954. 10.1021/acs.chemrev.7b00430 29190069

[B42] Zheng Y. Zheng L. Zhan Y. Lin X. Zheng Q. Wei K. (2007). Ag/ZnO Heterostructure Nanocrystals: Synthesis, Characterization, and Photocatalysis. Inorg. Chem. 46, 6980–6986. 10.1021/ic700688f 17658874

[B43] Zhou X. Wang S. Zhang C. Lin Y. Lv J. Hu S. (2021). Colorimetric Determination of Amyloid-β Peptide Using MOF-Derived Nanozyme Based on Porous ZnO-Co3O4 Nanocages. Microchim Acta 188, 56. 10.1007/s00604-021-04705-4 33502585

[B44] Zhu Y.-C. Zhang N. Ruan Y.-F. Zhao W.-W. Xu J.-J. Chen H.-Y. (2016). Alkaline Phosphatase Tagged Antibodies on Gold Nanoparticles/TiO2 Nanotubes Electrode: A Plasmonic Strategy for Label-free and Amplified Photoelectrochemical Immunoassay. Anal. Chem. 88, 5626–5630. 10.1021/acs.analchem.6b01261 27150939

