# Supplementary material for: Bioinspired Synthesis of ZnO@polydopamine/Au for Label-Free Photoelectrochemical Immunoassay of Amyloid-β Protein
Source: Front Bioeng Biotechnol. 2021 Nov 16;9:777344. doi: 10.3389/fbioe.2021.777344 (PMC8637201; doi:10.3389/fbioe.2021.777344)
Supplement: Supplementary file 1 [file DataSheet1.docx]

**Supplementary Information**

**Bioinspired synthesis of ZnO@polydopamine/Au for label-free photoelectrochemical immunoassay of amyloid-β protein**

Guangli He^a^, Yue Zhou^a^, Mifang Li^b^, Yanzhen Guo^a^, Hang Yin^a^, Baocheng Yang^a*^, Shouren Zhang^a^* Yibiao Liu^b^*

^a^ Henan Key Laboratory of Nanocomposites and Applications, Institute of Nanostructured Functional Materials, Huanghe Science and Technology College, Zhengzhou 450006, China.

^b^ Shenzhen Longgang Central Hospital (The Second Affiliated Hospital of the Chinese University of Hong Kong (Shenzhen), Shenzhen 518116, P.R. China.

E-mail: liuyibiao12345@126.com; shourenzhang@infm.hhstu.edu.cn; baochengyang@infm.hhstu.edu.cn


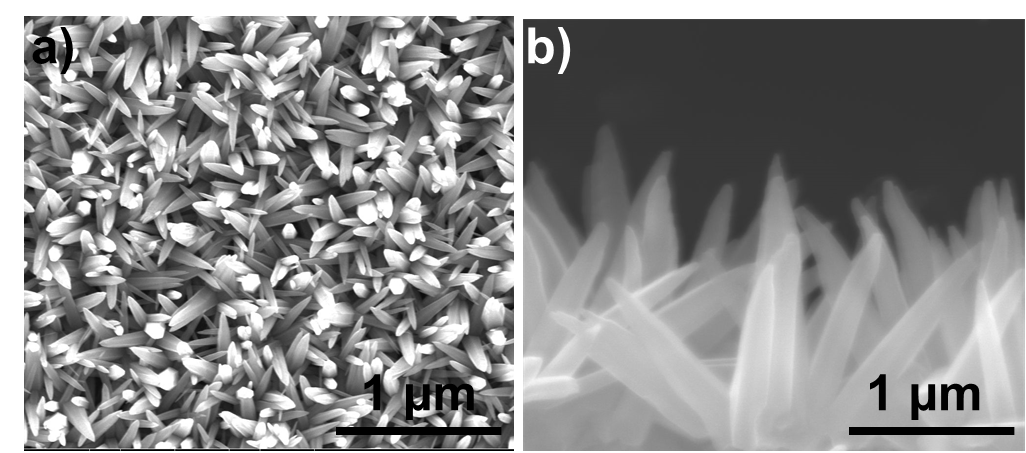


**Fig. S1** SEM images of ZnO NRs/ITO (a) top-view, (b) cross-view.


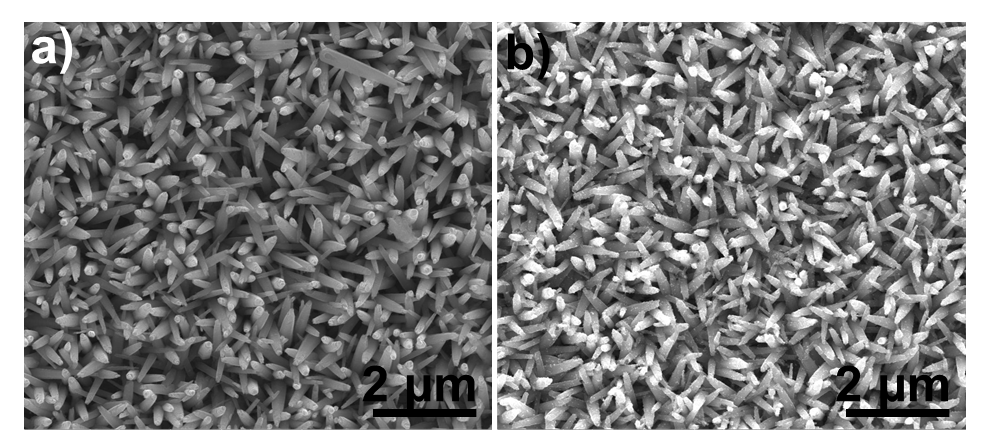


**Fig. S2** Low magnification SEM images of (a) ZnO@PDA and (b) ZnO@PDA /Au.

**Fig. S3** Photo image of (a) ZnO NRs/ITO, (b) ZnO@PDA, (c) ZnO@PDA /Au.


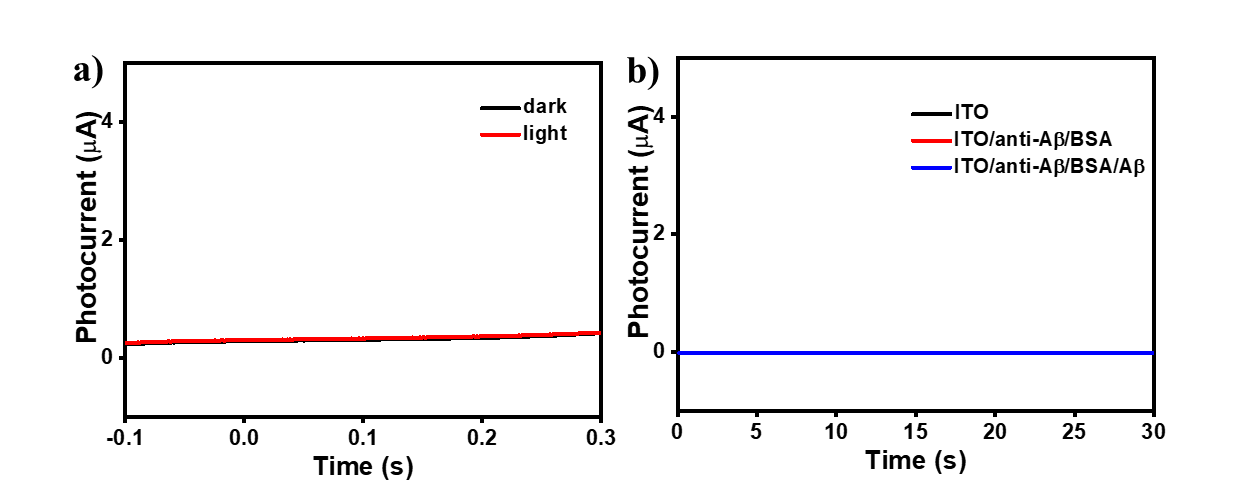


Fig. S4. (a) LSV curves of ITO without/with simulated sunlight illumination, respectively. (b) Photocurrent response of ITO, ITO/anti-Aβ/BSA, ITO/anti-Aβ/BSA/Aβ towards on/off cycles of simulated sunlight illumination at 0.1 V. Electrolyte solution was 0.1 M PBS containing 1 mM ascorbic acid, potential sweep rate at 50 mV﹒s^−1^.


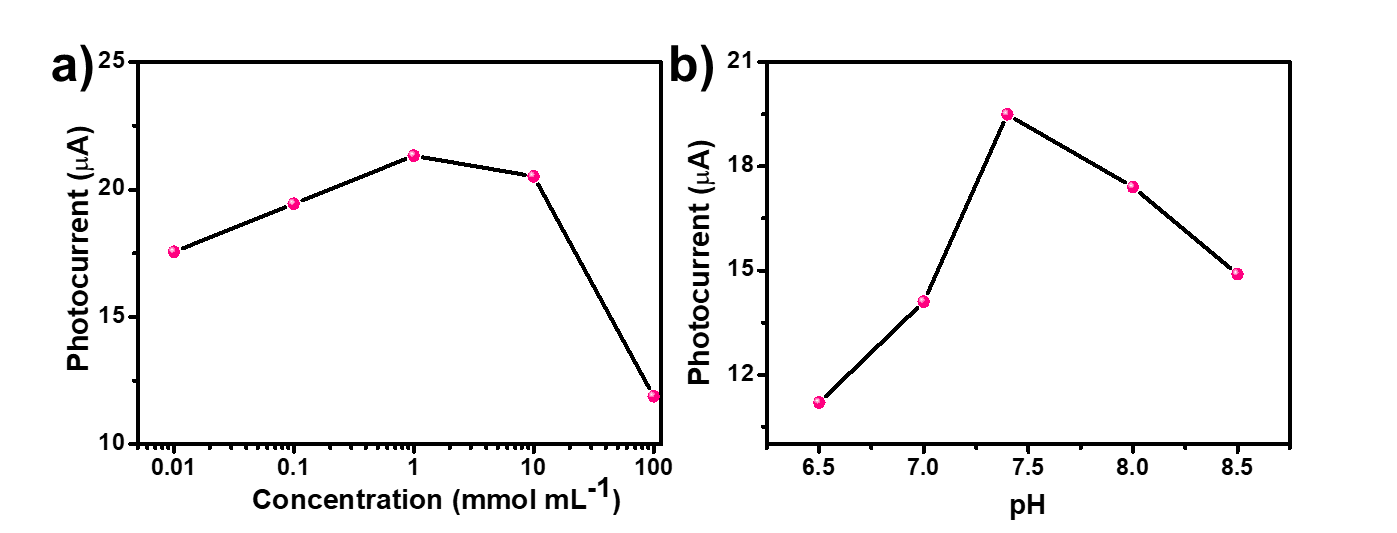


**Fig. S5** Effects of the AA concentration (a) and pH (b) on the performance of the immunosensor.

Fig. S6. Effect of the concentration of Aβ42 antibody immobilized on the ZnO@PDA/Au electrode for the detection of 100 ng/mL Aβ.

**Fig. S7** The photocurrent response of different labels:(a) ZnO@PDA /Au, (b) ZnO@PDA /Au/anti-Aβ, (c) ZnO@PDA /Au/anti-Aβ/BSA, (d) ZnO@PDA /Au/anti-Aβ/BSA/Aβ.


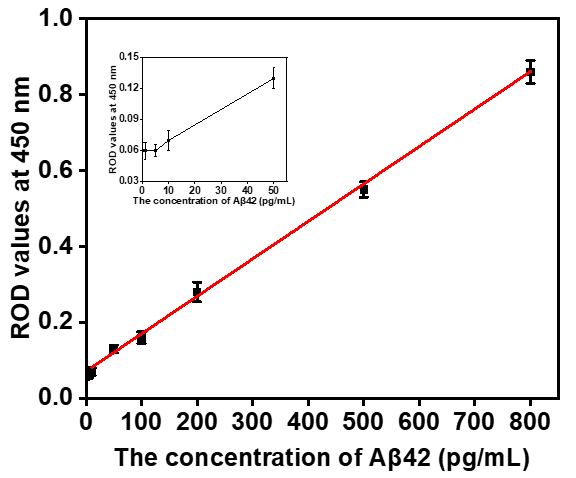


**Fig. S8** ELISA calibration curve of Aβ42 for cross validated the PEC sensor determination of Aβ42. The ELISA calibration curve was constructed by plotting the optical density of Aβ42 at 450 nm (TMB as substrate). And the range of Aβ42 concentration is 1-800 pg/mL. The relationship between the optical density at 450 nm and the Aβ42 concentration follows the regression equation y = 0.00099x + 0.072 (R^2^ =0.9991). The detection limit is 18.59pg/mL, and the linear range is from 10 pg/mL to 800 pg/mL.


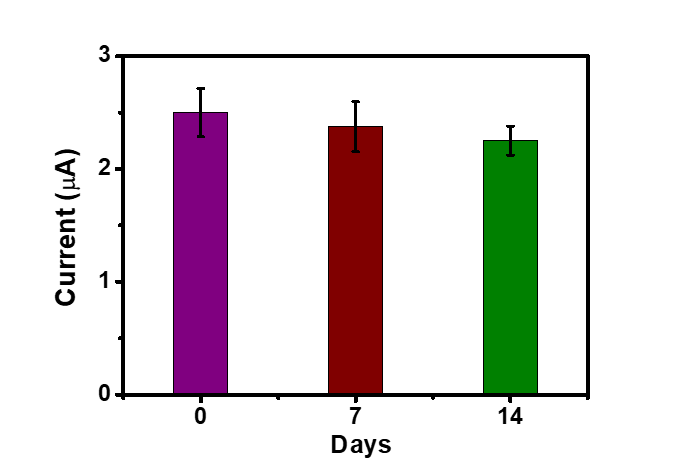


**Fig. S9** Stability of the immunosensor after storage at 4 ^o^C for 1 day, 7 days and 14 days, Aβ concentration is 10 ng/ml.

Fig. S10. Comparison of the photocurrent response of ZnO@PDA/Au electrode prepared in three batches.

**Table S1** Comparison between the proposed PEC sensor and other sensors for amyloid β-protein.

| Method | Materials | Linear range | Detection limit | References |
| --- | --- | --- | --- | --- |
| Electrochemical immunosensor | CNT-CuO | 1 – 66 ng/mL | 0.4 pg/mL | (Moreira et al. 2018) |
| Electrochemical immunosensor | Cu- Au-VG/CC | 0.01 – 2.2 nM | 3.5 pM | (Zhou et al. 2021) |
| SERS | PS@Au@MBA-Al^3+^ | / | 0.1 µM | (Guerrini et al. 2015) |
| Fluorescence detection | Cu^2+^-gCNQD | 1 – 700 ng/mL | 0.18 ng/mL | (Yin et al. 2019) |
| Colorimetric immunosensor | Cellulose | 1 – 10000 ng/mL | 710 pg/mL | (Moreira et al. 2021) |
| ELISA | Maxisorp microwell plates | / | 390 pg/ml | (Song et al. 2020) |
| PEC sensor | ZnO/Ru(bpy)_3_^2+^/Ce-CdS- polystyrene@CuS | 0.001 – 100 ng/mL | 0.37 pg/mL | (Fan et al. 2019) |
| PEC sensor | ZnO@PDA/Au | 0.001 – 100 ng/mL | 0.26 pg/mL | This work |

**Table S2** Recovery study for detecting amyloid β-protein in diluted goat serum.

| C_added_ (pg/mL) | C_founded_ (pg/mL) | Recovery (%) |
| --- | --- | --- |
| 1 | 1.025 | 102.5 |
| 10 | 9.71 | 97.1 |
| 100 | 104.83 | 104.8 |
| 1000 | 1074.1 | 107.4 |
| 10000 | 10913.7 | 109.1 |

**Reference**

[1] Moreira, F., Rodriguez, B., Dutra, R., Sales, M. Redox probe-free readings of a β-amyloid-42 plastic antibody sensory material assembled on copper@carbon nanotubes. 2018, Sensors & Actuators B Chemical, 264, 1-9. DOI: 10.1016/j.snb.2018.02.166

[2] Zhou Y, Lv Y, Dong H, Liu L, Mao G, Zhang Y, Xu M. Ultrasensitive assay of amyloid-beta oligomers using Au-vertical graphene/carbon cloth electrode based on poly(thymine)-templated copper nanoparticles as probes. Sensors & Actuators B Chemical, 2021, 331, 129429. DOI：10.1016/j.snb.2020.129429

[3] Guerrini, L., R. Arenal, B. Mannini, F. Chiti, R. Pini, P. Matteini, and R. A. Alvarez Puebla. SERS detection of amyloid oligomers on metallorganic-decorated plasmonic beads. ACS Appl. Mater. Interfaces, 2015, 7, 9420–9428. DOI: 10.1021/acsami.5b01056

[4] Yin Z, Si M, Ding J, et al. Transition metal-coordinated graphitic carbon nitride dots as a sensitive and facile fluorescent probe for β-amyloid peptide detection. Analyst, 2019, 144, 504-511. DOI: 10.1039/C8AN01620H

[5] Moreira F. T. C., Correia B. P., Sousa M. P., Sales, G. F. Colorimetric cellulose-based test-strip for rapid detection of amyloid β-42. Microchimica Acta, 2021, 188, 1-10. DOI：10.1007/s00604-021-04996-7

[6] Fan D, Liu X, Bao C, et al. A novel sandwich-type photoelectrochemical immunosensor based on Ru(bpy)32+ and Ce-CdS co-sensitized hierarchical ZnO matrix and dual-inhibited polystyrene@CuS-Ab2 composites. Biosensors and Bioelectronics, 2019, 129, 124-131. DOI: 10.1016/j.bios.2019.01.029

[7] Zhang N, Wang Y, Zhao G, Wang C, Li Y, Zhang Y, Wang Y, Wei Q. Photoelectrochemical immunosensor based on CdS/CdTe co-sensitized SnO2 as platform for ultrasensitive detection of amyloid β-protein. Analyst, 2019, 145, 619-625. DOI：10.1039/C9AN01848D
